# Systematic Evaluation of Competing Brain Transcriptomic Representations Reveals Reciprocal Patterns Across Heterogeneous Contexts

**DOI:** 10.3390/ijms27136083

**Published:** 2026-07-07

**Authors:** Zongnan Lyu, Chunxue Shao, Qi Yu, Renyu Yang, Guang Yang, Ziheng Wang

**Affiliations:** Division of Computational Biology, Chinese Center of Exercise Epidemiology, Northeast Normal University, Changchun 130024, China

**Keywords:** brain transcriptomics, direction classes, intervention-like contrasts, adversity-like contrasts, reciprocal representation, cross-study integration, hippocampus

## Abstract

Adaptive and adverse brain states are often assumed to lie on a shared molecular continuum, but this assumption has rarely been evaluated against explicit transcriptomic alternatives. This study aimed to compare two representations of cross-context brain transcriptomic organization: a transcriptome-wide global-axis model and a low-dimensional reciprocal model. We benchmarked these models across a curated cross-study brain cohort spanning exercise, alcohol-related adversity-like contexts, stress, aging, and neurodegeneration, using prespecified intervention-like and adversity-like directional contrast labels rather than assuming homogeneous biological states. We assessed the competing representations using signed-effect correlations, permutation analyses, non-linear fitting, and held-out reconstruction, and we then examined the resulting structure through region-specific human bulk evaluation and exploratory cellular, single-nucleus, spatial, and chromatin projection analyses. These downstream analyses were used to examine localization and biological interpretability and were not treated as independent evaluation of the module 1/module 2 (M1/M2) partition. The combined signed-effect statistics were interpreted as representation-level directional summaries rather than estimates of a homogeneous cross-study biological effect. The global-axis model received limited support: intervention-like and adversity-like signed-effect summaries were only weakly correlated, were not stronger than permutation null expectations, and were not improved by non-linear fitting. Within the selected reciprocal-gene space, a rank-1 latent profile reconstructed held-out genes more accurately than the hard M1/M2 partition, whereas the M1/M2 discretization provided a more interpretable but selection-conditioned directional summary. Human analyses yielded an asymmetric pattern: a significant M1 association was observed only in the hippocampal dataset, whereas M2, the reciprocal index, and the other examined brain regions showed no consistent corresponding effects; leave-one-stratum-out analyses indicated poor cross-stratum reproducibility of the exact gene-level partition. These findings motivate a low-dimensional reciprocal representation as an exploratory framework while emphasizing context dependence, cohort dependence, and heterogeneity.

## 1. Introduction

Brain adaptation and adversity-associated brain states are usually studied as separate biological problems. Exercise is commonly analyzed as a protective or restorative intervention [1,2,3,4], whereas alcohol exposure, chronic stress, and neurodegenerative states are often studied within disease-specific frames [5,6,7]. Yet these conditions perturb overlapping neural systems [8,9], raising a broader question: when the brain is pushed towards adaptive versus adverse states, is the shared transcriptomic organization best described as a single continuum or as a more structured relationship [10,11]?

We use three terms in this study in a deliberately operational sense. A transcriptome-wide “single-dial” hypothesis would predict that many genes shift in one direction in intervention-like contrasts and in the opposite direction in adversity-like contrasts; we refer to this hypothesis as the *global-axis model*. A structured-subspace alternative would predict that opposite behavior is concentrated in a smaller coordinated subset of genes rather than distributed across the transcriptome; we refer to this alternative as the *reciprocal representation*. A *direction class* is only a bookkeeping label used to orient heterogeneous study contrasts before comparison; it places each contrast on an intervention-like or adversity-like side without claiming that either side is biologically homogeneous.

This is fundamentally a representation problem rather than a standard differential-expression problem [12]. Differential expression can identify genes that change within one experiment, but it does not by itself determine how multiple heterogeneous experiments should be organized relative to one another [13,14]. In cross-context studies, the key object is not simply the list of significant genes from any one dataset, but the representation that best summarizes how operationally oriented intervention-like and adversity-like contrasts relate across species, regions, platforms and perturbation classes [15,16,17]. Without making that representation explicit, broad claims about intervention-like effects, reversal, or adversity-like effects remain difficult to compare and easy to overstate.

The global-axis model corresponds to one widely assumed representation, in which adaptive and adverse states occupy opposite ends of a transcriptome-wide continuum [18,19]. This idea is attractive because it is simple, continuous, and easy to map onto intuitive narratives of reversal or protection. But it remains a testable model, not a default truth. In a heterogeneous brain setting, the global-axis model can fail for at least two reasons: the shared signal may be weak relative to study-specific variance [20,21], or it may be concentrated in a restricted low-dimensional structure that does not align with one transcriptome-wide direction [22,23]. Testing the global-axis model therefore requires an explicit comparison against alternatives rather than treating axis-like opposition as the null biological expectation.

The reciprocal representation corresponds to the alternative possibility that the shared signal occupies a structured low-dimensional subspace [24]. Under this view, intervention-like and adversity-like contrasts are not mirror images across the whole transcriptome, but they are coupled through a lower-dimensional organization in which counter-directed behavior is concentrated in a subset of coordinated profiles [25,26]. Such a representation solves two conceptual problems at once. It allows the shared structure to be weaker than a transcriptome-wide continuum while still being biologically meaningful, and it separates held-out reconstruction from structural interpretability: a rank-1 latent profile model may provide more compact reconstruction, whereas a coarse reciprocal-gene discretization may provide a more interpretable but selection-conditioned directional summary [27,28,29].

Public transcriptomic resources now make this comparison possible, but only with explicit attention to heterogeneity [30]. Cross-study integration in the brain combines multiple species, brain regions, assay platforms, and perturbation designs, so integrated summaries cannot automatically be interpreted as one homogeneous biological effect [31,32,33]. They are better viewed as representation-level summaries of shared organization unless heterogeneity is modeled directly. This means that the relevant question is not simply whether any cross-study signal exists but which competing representation best captures the part of that signal that remains interpretable after harmonization and robustness analysis [34,35].

These definitions guided the contrast orientation used below. Exercise-related discovery contrasts were oriented toward the intervention-associated condition, whereas alcohol-, stress-, and pathology-related contrasts were oriented toward the exposure- or challenge-associated condition. These prespecified direction classes were used to formulate a testable representation-level hypothesis; they were not intended to define homogeneous biological states or to imply shared mechanisms within either class. The substantial heterogeneity among contexts was retained at the dataset and context levels and was evaluated through permutation, context-stratified summaries, leave-one-context-out analyses, and covariate-adjusted hierarchical models.

Here we assembled a curated cross-context cohort of public brain transcriptomic datasets spanning exercise, alcohol-related adversity-like contexts, stress, aging and neurodegeneration, and we analyzed the primary bulk component using a harmonized sample-level workflow. We treated the study as a benchmark of the two competing transcriptomic representations defined above: the global-axis model versus the reciprocal representation. We then asked four linked questions. First, does the global-axis model explain the shared signal in the original intervention-like versus adversity-like signed-effect summary space? Second, if not, does a structured low-dimensional subspace emerge under an operational low-dimensional approximation? Third, is that structure robust to threshold choice, heterogeneity adjustment, and hierarchical modeling? And fourth, does it remain interpretable when projected into human, cellular, spatial, and chromatin layers? The downstream human, cellular, spatial, and chromatin analyses were designed to evaluate region-specific associations and biological localization of fixed scores. They were not used to redefine the module 1/module 2 (M1/M2) partition, and these downstream layers were not interpreted as independent evidence for reproducibility of that partition. Under this design, the study does not claim one uniquely optimal decomposition. Rather, it identifies a structured, non-axis-aligned low-dimensional subspace that is not reducible to a single transcriptome-wide direction in the original summary space and that provides a more interpretable representation of cross-context brain transcriptomic organization in a heterogeneous setting.

## 2. Results

The Results are presented according to the analytical hierarchy defined above. We first describe the primary cross-study directional summaries and benchmark the global-axis and reciprocal representations. We then report robustness, heterogeneity, reconstruction, and reproducibility analyses of the selection-conditioned M1/M2 partition. Finally, we distinguish region-specific human bulk evaluation from exploratory cellular, spatial, pathway, and chromatin projection or contextualization analyses.

### 2.1. Primary Discovery Defines Cross-Study Directional Summaries

We first assembled a primary bulk discovery cohort restricted to datasets that passed sample-level differential-expression analysis, could be harmonized to a shared gene–symbol layer, and belonged to prespecified intervention-like or adversity-like contexts. This final discovery set comprised 13 primary bulk brain transcriptomic accessions spanning exercise–aging, exercise–stress, and exercise–neurodegeneration paradigms together with alcohol dependence, alcohol binge, alcohol preference, and glucocorticoid/stress paradigms assigned to the adversity-like direction class across mouse and rat brain tissue. An exercise-trait dataset was retained as supporting context, but excluded from primary estimation. Because these studies span multiple species, brain regions, and perturbation classes, the discovery analysis was designed to recover shared representational structure across heterogeneous contrasts rather than to estimate one common effect size.

Cross-study integration recovered a consistent gene-level directional signal under this stricter design. We mapped platform-specific features to harmonized gene symbols using local National Center for Biotechnology Information (NCBI) gene2ensembl, species-specific gene_info tables, and Gene Expression Omnibus (GEO) platform annotation, retaining a high proportion of analyzable features in most datasets, including 90.8% for GSE111212 [36], 98.9% for GSE164798 [37], 98.4% for GSE203554 [38] and 98.6% for GSE159136 [39]. Rather than selecting the most extreme probe or transcript per gene, we collapsed all mapped features within each dataset and combined signed effects across studies using effective-sample-size weighting. The leading genes in this representation-level ranking included *Ndufa10* (*n*_datasets_ = 11, combined signed *z*-score =6.07, false discovery rate (FDR) = $1.82\times 10^{-5}$), *Dnah12* (*n*_datasets_ = 7, combined signed *z*-score =−5.75, FDR = $1.03\times 10^{-4}$), *Nemf* (*n*_datasets_ = 12, combined signed *z*-score =5.67, FDR = $1.31\times 10^{-4}$), *Pcdh12* (*n*_datasets_ = 8, combined signed *z*-score =−5.53, FDR = $2.03\times 10^{-4}$), *Fzd10* (*n*_datasets_ = 8, combined signed *z*-score =−5.42, FDR = $3.42\times 10^{-4}$), and *Cdkn1c* (*n*_datasets_ = 12, combined signed *z*-score =−5.27, FDR = $6.59\times 10^{-4}$). These FDR values were used to prioritize genes within the weighted signed-effect aggregation and should not be interpreted as random-effects meta-analytic evidence for one shared biological effect across all datasets.

This integrated signal was distributed across studies rather than dominated by a single source. In leave-one-dataset-out analyses, the rerun cross-study directional summaries remained highly concordant with the full aggregation, with Spearman correlations ranging from 0.925 to 1.000 and top-100 overlap ranging from 52 to 100 genes. Removal of GSE31705 [40] produced the largest reduction in concordance (ρ=0.925), whereas removal of GSE29075 [41] had essentially no effect on rank order (ρ=1.000). Leave-one-context-out analyses gave similarly stable results, with Spearman correlations of 0.904 to 0.978 relative to the full aggregation; the largest reduction followed exclusion of the alcohol-dependence context (ρ=0.904), whereas exclusion of stress–glucocorticoid had a comparatively modest effect (ρ=0.978). These analyses support the presence of cross-study directional signal under the chosen aggregation scheme, while also indicating that heterogeneity is non-negligible and partly context-dependent.

### 2.2. Representation Benchmarking and Robustness Analyses Define the Reciprocal-Gene Space

We next performed the main benchmarking step of the study by comparing two operational descriptions of the relationship between intervention-like and adversity-like directional summaries. Under the global-axis model, the combined signed *z*-score profiles should show broad inverse alignment across shared genes. Across 17,766 shared genes, however, the profiles were only weakly correlated (ρ=0.101), and this relationship was not stronger than expected under random reassignment of datasets to intervention-like and adversity-like groups (empirical *p* = 0.515). These data do not support the transcriptome-wide global-axis representation in the original intervention-like versus adversity-like summary space.

We therefore evaluated an alternative reciprocal representation by focusing on genes with strong counter-directed behavior under the current ranking rule. Clustering of the top 100 reciprocal genes by dataset-level signed *z*-score profiles identified a two-module solution that provided a coarse M1/M2 discretization of the selected reciprocal-gene space. The smaller 19-gene partition, M1, showed higher signed summaries in intervention-like datasets and lower signed summaries in adversity-like datasets (mean combined signed *z*-score =2.73 in intervention-like summaries and −2.57 in adversity-like summaries). By contrast, the larger 81-gene partition, M2, showed the opposite pattern (mean combined signed *z*-score =−3.54 in intervention-like summaries and 2.08 in adversity-like summaries). Representative M1 genes included *Adgrl4*, *Zfp40*, *Aim2*, *Serinc3* and *Mpeg1*, whereas representative M2 genes included *Wfikkn2*, *Slc2a12*, *Togaram2*, *Adamtsl4*, and *Id1*. Because M1 and M2 were defined after reciprocal-gene selection, we use them as a selection-conditioned descriptive partition rather than as transcriptome-wide modules identified independently of the selection step. Their opposite directions are partly induced by the selection rule and therefore are not treated as independent evidence for reciprocal organization.

The selection-conditioned partition was nevertheless useful for summarizing context-level directional concentration. M1 was positively enriched in exercise–aging (z=2.44), exercise–stress (z=8.15) and exercise–neurodegeneration (z=6.36), and negatively enriched in alcohol dependence (z=−5.70), alcohol binge (z=−4.53), and alcohol preference (z=−8.50), but not in stress–glucocorticoid. M2 showed the reciprocal pattern and extended more broadly across adversity-like contexts, including stress–glucocorticoid (z=11.45); all context-level empirical FDR values were 0.001. These context-level shifts help characterize the selected reciprocal-gene space, but they do not by themselves establish a selection-independent or reproducible two-module biological program.

Additional formal checks clarified both the limits of this discretization and the main interpretive contribution of the analysis. Across all shared genes, a non-linear locally estimated scatterplot smoothing (LOESS) model did not improve five-fold out-of-sample prediction of the adversity-like signed *z*-score from the intervention-like signed *z*-score relative to a simple linear model (mean root-mean-square error (RMSE) 1.0233 versus 1.0232), arguing against an obvious hidden one-dimensional non-linearity in the same summary space. Within the 13-dimensional dataset-profile space used to define the reciprocal genes, however, the first principal component explained only 37.7% of variance, indicating that a single dominant profile axis did not capture most of the top reciprocal-gene profile matrix. A stricter head-to-head comparison within this profile space showed that the rank-1 latent profile reconstructed held-out genes more accurately than the hard two-centroid M1/M2 model (five-fold out-of-sample RMSE 0.644 versus 0.665). Thus, the M1/M2 partition does not have a reconstruction advantage over the simplest latent-profile alternative. Its value is interpretive: it provides a directionally explicit summary that can be visualized and projected into context-level, cell-type, spatial, and chromatin analyses. Module-definition sensitivity analyses showed that silhouette analysis selected k=2 in all combinations of reciprocal-gene cutoffs from 50 to 150 and linkage methods from Ward to complete linkage. Relative to the reference two-module solution, mean Jaccard overlap for the M1 and M2 partitions ranged from 0.47 at the top-50 cutoff to 0.96 at the top-100 cutoff. Bootstrap resampling of discovery datasets revealed a more restrictive picture: reference genes were selected only intermittently (median selection frequency 0.27 for M1 and 0.26 for M2). When selected, they were assigned to the same directional side with near-perfect consistency, but this conditional assignment consistency applies only to genes that re-entered the selected set. These analyses indicate that the coarse two-direction geometry was conditionally consistent under several internal perturbations, whereas exact gene selection and membership were unstable and dependent on the discovery cohort.

We next performed the principal cross-species robustness analysis by repeating the complete representation analysis after restriction to high-confidence, bidirectionally unique one-to-one mouse–rat orthologs. Ensembl BioMart release 116 provided 14,559 eligible ortholog pairs. After application of the original accession-level differential-expression estimates and cross-study aggregation procedure, 13,740 genes remained in the mapped joint gene space, representing 77.3% of the original 17,766-gene space.

The ortholog-restricted reciprocal scores were highly concordant with the original scores (Spearman ρ=0.996). Eighty-six genes were shared between the original and ortholog-restricted top-100 reciprocal-gene sets, corresponding to a Jaccard index of 0.754. The ortholog-restricted solution contained 17 M1 and 83 M2 genes, compared with 19 M1 and 81 M2 genes in the original analysis. Among the 86 genes shared between the two top-100 sets, all 15 shared M1 genes and all 71 shared M2 genes retained their original assignments. No shared gene switched between M1 and M2, yielding an adjusted Rand index of 1.00.

**Figure 1:**
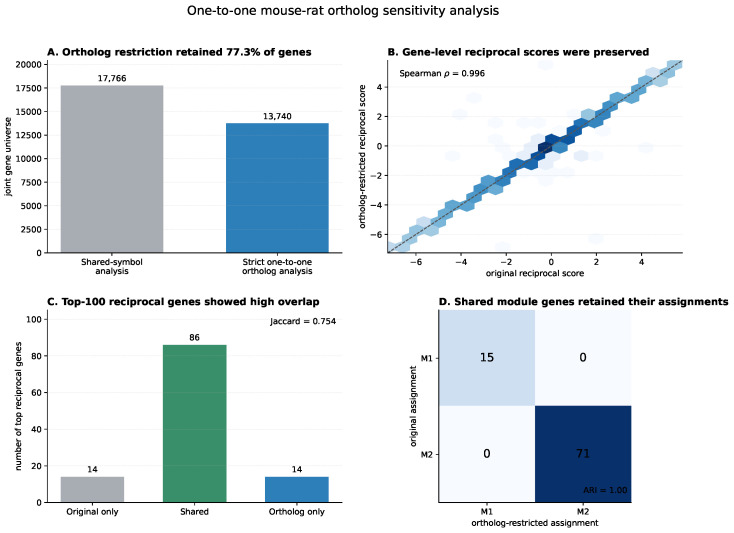
Robustness of the reciprocal-gene representation to strict one-to-one mouse–rat ortholog restriction. (**A**) Comparison of the original gene–symbol-harmonized joint gene space with the gene space retained after restriction to high-confidence, bidirectionally unique one-to-one orthologs. (**B**) Concordance of reciprocal scores among genes represented in both the original and ortholog-restricted analyses. (**C**) Overlap between the original and ortholog-restricted top-100 reciprocal-gene sets. (**D**) Correspondence of module 1 (M1) and module 2 (M2) assignments among the 86 genes shared between both top-100 sets. All 15 shared M1 genes and all 71 shared M2 genes retained their original assignments. Orthology relationships were obtained from Ensembl BioMart release 116 and restricted to ortholog_one2one records with an orthology-confidence score of 1 and bidirectionally unique official gene–symbol relationships.

The transcriptome-wide intervention-like versus adversity-like correlation remained weak after ortholog restriction (ρ=0.090, compared with ρ=0.101 in the original analysis) and was not significant under 1000 dataset-label permutations (empirical p=0.667). Within the ortholog-restricted top-100 reciprocal-gene space, the average silhouette width was 0.400 for k=2, compared with 0.256 for k=3 and 0.208 for k=4. Thus, strict removal of ambiguous, non-one-to-one, and species-specific relationships did not materially change the representation-level conclusion or the conditional two-direction geometry of the selected reciprocal-gene space. However, the incomplete top-100 overlap also supports our more limited interpretation that exact gene selection remains dependent on the analyzed feature universe and discovery cohort (Figure 1).

We then asked whether reciprocal alignment survived explicit modeling of structured heterogeneity and whether the exact coarse M1/M2 partition reproduced across major strata. In approximate random-effects meta-regression on dataset-level M1 and M2 scores, the directional contrast remained positive for M1 and negative for M2 after adjustment for species, platform, and hippocampal versus non-hippocampal status. After additional adjustment for a composition-derived principal component and a generic-stress proxy, the M1 estimate remained directionally positive but became imprecise (β=1.06, *p* = 0.160). The M2 estimate remained strongly negative (β=−2.45, *p* = 0.0012). Weighted fixed-effect models gave the same qualitative result. However, leave-one-stratum-out analyses showed poor cross-stratum reproducibility of the exact partition. Training sets that excluded mouse, rat, ribonucleic acid (RNA) sequencing (RNA-seq), or non-hippocampal strata did not yield an estimable two-module solution under the current operating rules. When the solution remained estimable, overlap with the reference partition was low (mean Jaccard 0.12 for microarray holdout and 0.15 for hippocampal holdout). Held-out directional concordance was also modest, at 0.50 and 0.40, respectively. These analyses indicate substantial cohort dependence and poor reproducibility of the exact M1/M2 partition across major structural strata.

**Figure 2:**
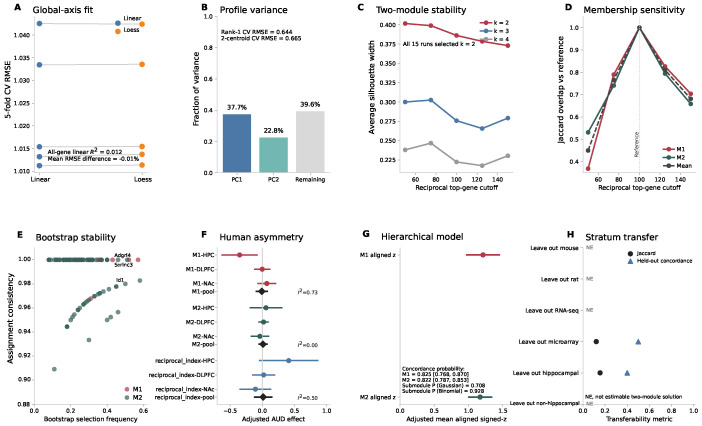
Reconstruction, conditional-consistency, and heterogeneity analyses for the reciprocal-gene representation. (**A**) Five-fold out-of-sample root-mean-square error (RMSE) for linear and locally estimated scatterplot smoothing (LOESS) models predicting the adversity-like combined signed *z*-score from the intervention-like combined signed *z*-score across all shared genes. (**B**) Variance decomposition of the dataset-profile matrix for the top 100 reciprocal genes, showing the fractions of variance explained by the first principal component (PC1), the second principal component (PC2), and the remaining components. Inset values report held-out reconstruction error for a rank-1 latent profile model and a hard two-centroid module 1/module 2 (M1/M2) model; lower RMSE indicates better reconstruction. (**C**) Average silhouette width across reciprocal top-gene cutoffs for candidate k=2, k=3, and k=4 cluster solutions. (**D**) Jaccard overlap of M1, M2, and the mean assignment relative to the reference top-100 solution across reciprocal top-gene cutoffs. (**E**) Bootstrap behavior of reference reciprocal genes, shown by bootstrap selection frequency and assignment consistency conditional on selection. (**F**) Adjusted alcohol use disorder (AUD)-effect estimates across bulk human datasets for M1, M2, and the reciprocal index, shown as coefficient estimates with 95% confidence intervals (CIs) together with inverse-variance pooled summaries. (**G**) Gene-by-dataset hierarchical mixed-model estimates after simultaneous adjustment for species, platform, region, and context. Points show covariate-adjusted marginal means on the aligned signed *z*-score scale with 95% CIs for M1 and M2, and inset text reports the corresponding sign-concordance probabilities from a parallel binomial mixed model. (**H**) Leave-one-stratum-out reproducibility of the coarse M1/M2 discretization. Filled circles show the mean Jaccard overlap with the reference partition and triangles show held-out directional concordance. Not estimable (NE) denotes settings in which the reduced training subset did not support an estimable two-module solution under the current operating rules.

We next asked whether the same aligned directional signal survived a more explicit hierarchical treatment of species, region, platform, and context. We fit a gene-by-dataset Gaussian mixed model to the reciprocal-gene matrix. The model included structural terms for M1/M2 partition label, species group, assay platform, region group, and biological context. It also included random intercepts for dataset and repeated gene identity. After simultaneous adjustment, the grand-mean intercept on the aligned signed *z*-score scale remained positive (intercept =0.85, *p* = $7.5\times 10^{-13}$). Covariate-adjusted marginal means on the same scale were similar for M1 and M2 (M1 =1.21, 95% confidence interval (CI) 0.97 to 1.45; M2 =1.17, 95% CI 1.00 to 1.34). The M1/M2 partition-label term itself was small (*p* = 0.708). A parallel binomial mixed model treating sign concordance as the outcome gave the same qualitative result. The adjusted predicted probability of directional agreement was 0.825 (95% interval 0.768 to 0.870) for M1 and 0.822 (0.787 to 0.853) for M2, with no detectable M1–M2 difference (*p* = 0.928). Species and context contributed more than platform or broad region in these models. Thus, aligned reciprocal-gene behavior persisted after coarse multilevel adjustment, but this result should be separated from reproducibility of the exact M1/M2 gene boundary, which remained unstable and poorly reproducible across strata. These reconstruction, conditional-consistency, and heterogeneity analyses are summarized in Figure 2.

### 2.3. Exploratory Reference-Atlas and Single-Nucleus Localization of Fixed Module Scores

Having defined a coarse M1/M2 discretization of the reciprocal representation, we next asked where it projected across major brain cell classes. In a preprocessed single-cell reference atlas collapsed to six broad cell classes, M1 projected most strongly to microglial profiles (relative z=2.07), whereas M2 projected most strongly to endothelial-associated profiles (relative z=2.21). These findings indicate non-uniform cellular localization at the broad cell-class level.

We then used GSE237885 as an exploratory hippocampal single-nucleus localization follow-up spanning wild-type (WT) and amyloid precursor protein (APP) transgenic backgrounds with and without exercise. Marker-based assignment identified excitatory, inhibitory, astrocytic, endothelial, microglial, and oligodendroglial populations across all four samples. Within this follow-up, endothelial nuclei repeatedly showed the highest projected scores for both modules, whereas microglia showed relatively elevated M1 scores compared with most neuronal and oligodendroglial classes. These analyses provide an exploratory cellular localization layer for the fixed M1/M2 scores, with modest and heterogeneous within-genotype exercise-related shifts (Supplementary Figure S1).

### 2.4. Exploratory Pathway Interpretation Highlights Signaling-Related Features

We next asked whether the M1/M2 discretization admitted interpretable pathway-like projections and signaling interfaces. Because M1 and M2 are relatively compact and were defined through a cross-species harmonized framework, this analysis focused on annotating projected themes within the reciprocal representation. The resulting annotations suggested that both modules contain plausible communication-related and signaling-interface genes, although statistical support after multiple-testing correction was generally modest. These pathway-like and signaling-interface annotations are summarized in Supplementary Figure S2.

### 2.5. Secondary Human Evaluation Reveals a Hippocampal Module 1 Association but No Consistent Cross-Region Pattern

Human analyses showed an asymmetric pattern rather than a consistent cross-region pattern. In GSE181804, a bulk human hippocampus dataset with alcohol use disorder (AUD) and control samples, M1 was significantly reduced in AUD in the base model (β=−0.326, *p* = 0.025). This effect was not attenuated after the addition of endothelial and generic-stress proxy scores; if anything, it became slightly stronger (β=−0.353, *p* = 0.021). By contrast, M2 alone showed the expected positive direction but was not significant, and the paired reciprocal index (M2 minus M1) showed a concordant but weaker AUD-associated increase in both the base and adjusted models (β=0.402, *p* = 0.082; adjusted β=0.409, *p* = 0.095). Thus, a significant human bulk association was observed only for hippocampal M1 and did not show consistent corresponding effects for M2, the reciprocal index, or the full reciprocal representation across brain regions.

**Figure 3:**
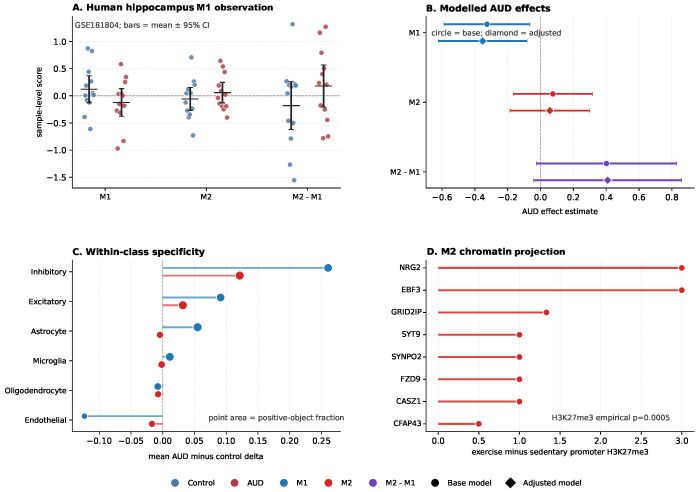
Hippocampal module 1 (M1) observation, specificity controls, and chromatin projection. (**A**) Sample-level M1, M2, and reciprocal-index (M2 minus M1) scores in the human hippocampus alcohol use disorder (AUD) dataset GSE181804. Black bars denote mean ± 95% confidence interval (CI). (**B**) AUD effect estimates for M1, M2, and the reciprocal index in GSE181804 before and after adjustment for endothelial and generic-stress proxy scores, shown as coefficient estimates with 95% CIs. (**C**) Mean AUD-minus-control shifts for M1 and M2 within broad cell classes across readable GSE277313 caudate objects. Labels indicate the fraction of objects showing a positive delta in that class. (**D**) Top M2 genes with exercise-associated promoter histone H3 lysine 27 trimethylation (H3K27me3) gain in the separate chromatin accession. The M2 partition showed significant enrichment for H3K27me3 alignment (empirical *p* = 0.0005), interpreted as chromatin projection rather than mechanistic inference. Panels A and B summarize the GSE181804 hippocampus dataset as a human hippocampal evaluation, panel C summarizes class-resolved shifts from GSE277313, and panel D shows promoter H3K27me3 alignment for M2.

Specificity-control analyses argued against simple vascular or generic-stress explanations for the human hippocampus effects. At the gene-content level, module overlap with the top endothelial marker set and with a curated immediate-early and heat-shock stress-response set was minimal; M2 overlapped only one endothelial marker out of 81 genes and no stress-response genes, whereas M1 showed limited microglial overlap but no endothelial or stress-gene overlap. At the sample level in GSE181804, M1 showed essentially no correlation with the endothelial proxy (ρ=0.011), and the AUD effect persisted after adjustment for both endothelial and stress proxies. Although latent composition effects may still contribute, these controls reduce the likelihood that the hippocampal human signal is merely a vascular or generic stress-response surrogate. A class-resolved summary from GSE277313 provided a specificity-control layer to probe composition-related alternatives. Figure 3 summarizes the hippocampal M1 observation together with specificity-control analyses.

### 2.6. Secondary Cross-Region Human Evaluation and Exploratory Single-Cell Projection Show Non-Uniform Patterns

A second bulk human dataset (GSE253155) showed weak and tissue-dependent effects on M1, M2, and the reciprocal index in the dorsolateral prefrontal cortex (DLPFC) and nucleus accumbens, with no significant AUD association before or after adjustment for endothelial and stress proxies. The human findings are therefore asymmetric and non-uniform: GSE181804 showed a hippocampal M1 association, whereas DLPFC, nucleus accumbens, and the caudate single-cell projections did not provide consistent cross-region evidence for the full reciprocal representation.

This asymmetry was also apparent when adjusted bulk-human effects were pooled across datasets. Inverse-variance pooling of hippocampus, DLPFC, and nucleus-accumbens effects gave pooled estimates close to zero for M1, M2, and the reciprocal index, with substantial heterogeneity for M1 ($I^{2}=0.73$) and more modest heterogeneity for the reciprocal index ($I^{2}=0.50$). Together, the pooled estimates argue against a uniform cross-region human effect and indicate that the observed human bulk association is concentrated in hippocampal M1.

We next asked whether the selection-conditioned M1/M2 scores could be projected into additional human AUD datasets. In GSE277313, a caudate-nucleus single-cell resource, nine readable objects retained both AUD and control cells for exploratory comparison. Across these objects, six showed higher overall projected scores in AUD than in control cells for both M1 and M2, indicating recurrent projection of the selection-conditioned scores in human AUD tissue. When the analysis was summarized within broad cell classes rather than across pooled cells, positive AUD-minus-control shifts were most reproducible in excitatory and inhibitory neurons for both modules, whereas endothelial shifts were inconsistent and on average negative. These exploratory object-level and class-resolved projections are summarized in Supplementary Figure S3.

### 2.7. Exploratory Chromatin Contextualization of the Adversity-like Side

We next asked whether either side of the reciprocal representation aligned with a separate molecular layer. In the exercise-related chromatin accession GSE208633, M2 showed significant enrichment for exercise-associated promoter histone H3 lysine 27 trimethylation (H3K27me3) gain (empirical *p* = 0.0005), whereas analogous alignment for M1 or for histone H4 lysine 8 acetylation (H4K8ac) was not detected. Gene-level inspection identified several M2 members with prominent positive H3K27me3 changes, including *EBF3*, *NRG2*, *GRID2IP*, *CASZ1*, *FZD9*, and *SYT9*. Because M2 is downregulated in intervention-like datasets and upregulated in adversity-like datasets, this alignment is consistent with a regulatory correlate for the adversity-like side of the reciprocal representation. The chromatin-alignment findings in Figure 3 provide a correlational projection and contextualization layer for the adversity-like side of the selection-conditioned partition, rather than mechanistic inference or a test of module reproducibility.

### 2.8. Exploratory Anatomical Spatial Localization in a Human Hippocampus Section

To add a coordinate-aware tissue-image spatial view of the reciprocal representation, we projected the fixed M1 and M2 modules into a representative Visium hippocampus section from GSE264692, a human single-nucleus and spatial transcriptomics atlas that provides the coordinate and histology metadata necessary for anatomical reconstruction. This representative section contained 4992 in-tissue spots after alignment to the supplied position file. Because GSE264692 was not designed as an intervention-like versus adversity-like comparison, this analysis served as an anatomical localization layer.

**Figure 4:**
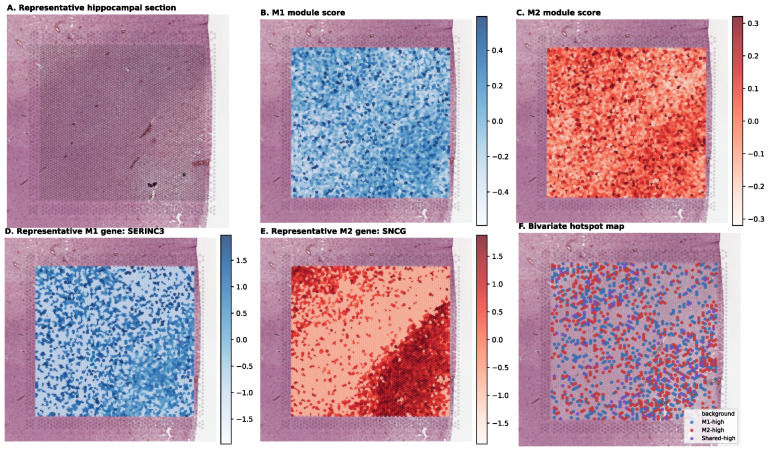
Coordinate-aware anatomical localization of module 1 (M1) and module 2 (M2) in a representative human hippocampus section from GSE264692. (**A**) Histology-aligned Visium section with the in-tissue spot footprint shown over the supplied tissue image. (**B**) Spatial map of the M1 module score from the selection-conditioned coarse M1/M2 discretization, after within-section gene-wise standardization. (**C**) Spatial map of the corresponding M2 module score in the same section. (**D**) Spatial expression pattern of *SERINC3*, the representative M1-tracking gene selected by within-section correlation with the M1 score. (**E**) Spatial expression pattern of *SNCG*, the representative M2-tracking gene selected analogously. (**F**) Bivariate hotspot map using the 85th percentile of each module score to classify spots as M1-high, M2-high, shared-high, or background. The figure uses the tissue-coordinate and histology metadata supplied with GSE264692 to present anatomical localization of projected M1 and M2 scores. Spatial scale was estimated from the 10× Genomics Visium spot diameter and the accompanying Space Ranger scale factors; 500 μm corresponds to approximately 130 pixels in the high-resolution tissue image.

Both modules showed structured, non-uniform spatial organization across the section. M1 formed broader territories with sharper local peaks (maximum within-section score 3.97), whereas M2 showed a smoother but still clearly patterned distribution (maximum within-section score 1.09). Using the 85th percentile of each module score to define hotspots, we classified 590 spots as M1-high, 590 as M2-high, and 159 as shared-high, indicating only partial overlap between module-rich territories. Gene-level overlays chosen directly from the section reinforced this distinction: *SERINC3* tracked M1-enriched regions, whereas *SNCG* tracked M2-enriched regions. Figure 4 therefore illustrates the relative within-section localization of fixed M1 and M2 scores; it did not test whether the original cross-study directions were reproduced.

### 2.9. Exploratory Spatial Follow-Up in the Exercise–Aging Dataset Shows Heterogeneous Local Remodeling

We next asked whether the M1 and M2 modules could be visualized in a spatial transcriptomic setting linked to the exercise–aging arm of the study. Using the fixed M1 and M2 modules, we analyzed GSE271564, a spot-based spatial transcriptomics dataset spanning old exercise, old control and young control mouse brain samples. The locally available GEO archive contained spot-by-gene matrices, feature annotations and barcode identities for all three samples, but not the tissue-coordinate or histology-image files required for anatomical reconstruction. The analysis was therefore performed at the barcode-matched spot level as exploratory spatial follow-up rather than an independent reproducibility test.

After pooled gene-wise standardization across all spots, old exercise showed a small downward shift in M1 relative to old control (mean Δ=−0.014 across barcode-matched spots) and a slight upward shift in M2 (mean Δ=0.006), with broad overlap in the spot-score distributions. Barcode-matched scatterplots nevertheless showed localized deviations from the identity line for both modules, indicating that exercise-related differences were spatially heterogeneous across array positions. At the gene level, the largest old exercise versus old control changes within module genes included lower spatial-average signal for *MAP2* and *RAP1GAP*, together with higher signal for *VIM*, *C1QTNF4* and *PRKCD*. Together, these spot-level distributions, barcode-matched comparisons, and gene-level shifts indicate localized exercise-related remodeling in this exploratory spatial follow-up and are summarized in Supplementary Figure S4.

## 3. Discussion

The central contribution of this study is to recast cross-context brain transcriptomic integration as a representation problem [12,16] and to state explicitly what the benchmark did and did not support. The weak transcriptome-wide association between the prespecified intervention-like and adversity-like summaries, the non-significant class-label permutation result, and the absence of improvement with non-linear fitting did not support a simple transcriptome-wide global inverse axis in the assembled cohort. This conclusion is specific to the heterogeneous benchmark analyzed here and should not be read as evidence that all adaptive and adverse brain biology are unrelated. It does, however, count against the simplest global-axis representation for these prespecified directional summaries. The evidence hierarchy was therefore primary discovery and representation benchmarking first, robustness and sensitivity analyses second, secondary region-specific human bulk evaluation third, and exploratory localization, projection, and contextualization layers fourth.

The reciprocal analysis identified a more restricted finding. Within a selected subset of counter-directed genes, the data showed a structured reciprocal-gene space that could be summarized in an interpretable M1/M2 form. However, this result did not establish the M1/M2 partition as a uniquely superior or most predictive model. A rank-1 latent profile reconstructed held-out genes more accurately than the hard two-module partition, whereas M1/M2 offered a more interpretable directional summary of the selected reciprocal-gene space. Its value is therefore explanatory and visual: it converts a selected continuous profile space into fixed scores that can be projected into cellular, spatial and regulatory layers [22,27,34].

This interpretability came with substantial limitations. Because M1/M2 was derived after reciprocal-gene selection, the partition was selection-conditioned and did not provide independent evidence for reciprocal organization. Exact gene membership showed low bootstrap selection frequency, and several leave-one-stratum-out analyses failed to recover an estimable two-module solution. When a solution remained estimable, overlap with the reference partition and held-out directional concordance were low. The present M1/M2 partition should therefore be viewed as cohort dependent and poorly reproducible across major structural strata, rather than as a fixed cross-study gene program or a biomarker set that can be applied unchanged across species, brain regions or platforms [25,26].

The intervention-like and adversity-like labels should also be interpreted as operational direction classes, not as evidence that the included contexts form a single mechanistic dichotomy. The discovery cohort combines multiple species, brain regions, assay platforms, perturbation types, durations, disease backgrounds and cellular compositions. The weighted cross-study signed *z*-score aggregation should therefore be read as a representation-level directional summary under heterogeneity rather than as a pooled biological effect estimate [14,30,31]. Context-stratified summaries, leave-one-dataset-out and leave-one-context-out analyses, permutation tests, and covariate-adjusted hierarchical models reduced the likelihood that one obvious structural confound fully explained the result, but they did not eliminate dataset-composition effects or prove a common latent pathway.

The human and downstream projection analyses further narrowed the interpretation. Human analyses did not provide consistent evidence for the full reciprocal representation across modules or brain regions. The clearest observation was restricted to M1 in the hippocampal dataset, whereas M2 was not significant, the reciprocal index did not reach the conventional 0.05 threshold, and corresponding effects were not detected in DLPFC or nucleus accumbens. Specificity controls made the simplest vascular or generic-stress explanations less likely, but bulk-tissue signals can still reflect coupled variation in cell abundance, cell state, regional sampling, and pathology stage, which the current study did not model jointly [42,43]. Cellular, spatial, and chromatin analyses increased biological interpretability by localizing fixed scores and contextualizing selected genes, but they did not independently establish reproducibility, transferability, or mechanism [34,44].

## 4. Materials and Methods

### 4.1. Study Design and Dataset Curation

This study used a curated cross-context collection of public brain transcriptomic datasets to compare two candidate representations of heterogeneous intervention- and adversity-related contrasts. Dataset search, prioritization, eligibility assessment, and accession-level analytical roles are described below and summarized in Table 1. The final analyses included 21 unique NCBI Gene Expression Omnibus (GEO; National Center for Biotechnology Information, Bethesda, MD, USA) accessions represented by 22 analysis rows, because GSE253155 was analyzed separately in DLPFC and nucleus accumbens. Thirteen accessions formed the primary bulk discovery cohort and were used to define the primary reciprocal-gene structure. The remaining eight accessions were assigned to secondary human evaluation or exploratory localization, projection, and contextualization roles.

The analytical workflow was organized into four levels. Primary discovery and representation benchmarking used the 13 bulk transcriptomic accessions to construct cross-study directional summaries, compare the global-axis and reciprocal representations, and derive the selection-conditioned M1/M2 partition. Robustness and sensitivity analyses evaluated the dependence of these results on dataset and context composition, gene-selection thresholds, clustering choices, resampling, and major structural sources of heterogeneity. Secondary human evaluation applied the fixed M1/M2 definitions and scoring directions to human bulk datasets without redefining module membership. Reference-atlas, single-cell, single-nucleus, spatial, pathway, and chromatin analyses were used as exploratory localization, projection, and contextualization layers. All custom computational workflows were implemented in R software (R Foundation for Statistical Computing, Vienna, Austria).

Before differential-expression aggregation and representation modeling, the direction of each eligible primary contrast was fixed in the analysis registry. Exercise–aging, exercise–stress, and exercise–neurodegeneration contrasts were oriented toward the exercise-associated intervention condition and assigned to the intervention-like direction class. Alcohol-dependence, binge-exposure, alcohol-preference, and stress–glucocorticoid contrasts were oriented toward the exposure- or challenge-associated condition and assigned to the adversity-like direction class. These labels were prespecified directional contrast classes for representation-level testing, not mechanistic or diagnostic classifications. Studies assigned to the same class were not assumed to share a pathway, cell type, effect magnitude, or underlying disease process.

Dataset and biological-context identities were retained throughout the analysis and were examined using context-level summaries, leave-one-dataset-out and leave-one-context-out analyses, and models adjusting for species, assay platform, brain-region group, and biological context. Dataset-specific contrast definitions, input assumptions, sample-matching rules, and analytical roles were prespecified in the analysis registry. The cross-study aggregation was therefore interpreted as a representation-level directional summary under heterogeneity, not as evidence that all included studies estimated a common biological effect or shared a single latent pathway.

### 4.2. Dataset Search, Selection, and Analysis Roles

Public GEO records were identified from NCBI GEO using searches that combined exercise-related terms (exercise, running, wheel, and physical activity) or alcohol-related terms (alcohol, ethanol, withdrawal, and dependence) with central nervous system terms, including brain, hippocampus, cortex, amygdala, striatum, and accumbens. A complementary search combined exercise terms with aging, Alzheimer disease, stress, depression, and pain terms. After merging the search results and removing duplicate accessions, 347 candidate GEO records were screened.

Candidate records were ranked using the GEO title, summary, organism, assay type, sample number, and sample annotations. Higher priority was assigned to brain-relevant records with an interpretable exercise-, alcohol-, aging-, stress-, or neurodegeneration-related design and reusable transcriptomic data. The 100 highest-ranked records underwent detailed manual review of study design, sample annotations, Supplementary Files, and data availability. Automated ranking was used only to prioritize screening and did not determine final eligibility.

Records were retained when they contained central nervous system samples, addressed a relevant biological context, supported a reconstructable comparison or predefined projection analysis, provided sufficient metadata, and supplied reusable sample-level or feature-level data. Records were excluded when they involved non-neural tissues, lacked an interpretable comparison, provided insufficient sample annotations, contained only summary differential-expression results, or lacked a reusable data representation.

A total of 21 unique GEO accessions contributed directly to the results reported in this study (Table 1). These accessions are represented by 22 analysis rows because GSE253155 was modeled separately in DLPFC and nucleus accumbens. Thirteen bulk transcriptomic accessions formed the primary discovery cohort and were included in the weighted cross-study analysis. The remaining eight accessions were assigned to secondary human evaluation or exploratory reference-atlas anchoring, single-nucleus localization, human single-cell projection, chromatin contextualization, and spatial localization or follow-up. Candidate records that did not contribute to a reported analysis are not counted as unique GEO accessions in this study.

### 4.3. Dataset-Level Preprocessing and Differential-Expression Analysis

Contrasts for the 13 primary bulk accessions were defined before analysis using a structured registry containing the input type, organism, brain region, biological context, comparison groups, sample-matching rules, metadata fields used for matching, and analysis role. Delimited count and expression matrices were parsed by identifying numeric sample columns and removing annotation columns; GEO series-matrix files were parsed from the embedded matrix block. Illumina (San Diego, CA, USA) non-normalized microarray matrices with alternating signal and detection-*P* columns were handled by retaining signal columns only and restoring GEO sample identifiers as column names. Blank feature rows and rows without quantitative measurements were removed before analysis.

Sample metadata were obtained either from the processed expression file or, when file-level sample labels were incomplete, from corresponding GEO series-matrix metadata accessed through GEOquery (Bioconductor Project, Buffalo, NY, USA) [45]. Samples were assigned to groups using prespecified rules applied to sample identifiers or selected GEO metadata fields. Samples that did not match either registered group were excluded from the corresponding contrast. When processed expression columns and GEO sample annotations could not be matched directly by identifier, the workflow attempted exact matching, unique substring matching, and order-preserving matching when the registry explicitly indicated that the processed matrix columns followed GEO sample order.

Integer-like count matrices were analyzed using DESeq2 (Bioconductor Project, Buffalo, NY, USA) [46]. Features were retained when they had at least 10 counts in at least two samples, and differential expression was estimated with a simple group design in which analysis_group was the only model term. The workflow specified limma-voom (Bioconductor Project, Buffalo, NY, USA) [47] for count matrices with fewer than six selected samples, although no successfully analyzed primary dataset met this condition. Pre-normalized RNA-seq and microarray matrices were transformed as $\log_{2}\left(x+1\right)$ and analyzed using limma (Bioconductor Project, Buffalo, NY, USA) [35] with empirical-Bayes variance moderation. Output tables retained feature identifiers, log_2_ fold changes, nominal *p* values, and adjusted *p* values where available.

No additional biological covariates, surrogate variables, batch terms, or cross-dataset expression-level batch correction were applied to the 13 primary accession-level models because comparable covariate and batch information was not consistently available across studies. Expression matrices were analyzed separately within each accession-specific analysis and were not pooled before differential-expression analysis. Cross-study heterogeneity was evaluated downstream using leave-one-dataset-out, leave-one-context-out, structural-covariate sensitivity, and hierarchical mixed-model analyses.

Evaluation, reference, localization, and projection accessions were processed according to their intended analytical roles. The GSE115746 reference profiles were used to derive broad cell-class expression anchors. The four GSE237885 single-nucleus matrices were normalized separately to log1p counts per 10,000 and used for broad cell-class localization. Readable case–control objects from GSE277313 were used for exploratory human cell-class projections. Human bulk accessions GSE181804 and GSE253155 were normalized by trimmed mean of M-values (TMM) scaling followed by log-counts per million (log-CPM) transformation and analyzed using sample-level linear models with the available demographic and technical covariates. The GSE208633 chromatin data were used to quantify exercise-minus-sedentary promoter-mark changes. Spatial count matrices were normalized to log1p counts per 10,000 before module scoring.

### 4.4. Gene-Identifier Harmonization and Cross-Study Integration

Because the discovery cohort combined rat and mouse RNA-seq datasets, normalized RNA-seq matrices, and rat microarray studies, feature identifiers were harmonized to a shared gene–symbol layer before cross-study integration. Rat and mouse Ensembl identifiers (EMBL-EBI, Hinxton, UK) were normalized by removing version suffixes and mapped to official symbols using a local NCBI resource bundle composed of gene2ensembl and species-specific gene_info tables for *Mus musculus* and *Rattus norvegicus*. Probe-based identifiers from GPL1355 platform annotation tables (Affymetrix Rat Genome 230 2.0 Array; Thermo Fisher Scientific, Waltham, MA, USA) were mapped using the platform annotation table. Symbol-like identifiers were further canonicalized against species-specific official symbols and synonym tables to reduce redundancy introduced by alias usage.

After mapping, each dataset was collapsed to one row per harmonized gene. Gene-level log_2_ fold change was summarized as the within-dataset median across mapped features, whereas nominal *p* values were recomputed from an internal signed *z*-score aggregation across all mapped features assigned to that gene. This strategy avoided selecting the most extreme probe or transcript as the representative gene-level effect.

Gene-level cross-study directional summaries were then computed from the harmonized differential-expression tables. For each dataset, signed *z*-scores were derived from nominal *p* values and log_2_ fold change direction. Cross-study aggregation used a weighted Stouffer-style combination of signed *z*-scores [48], with dataset-specific weights proportional to the square root of the effective two-group sample size, $n_{\mathrm{eff}}$, where $n_{A}$ and $n_{B}$ denote the sample sizes of the two comparison groups:

$$\begin{array}{cc} n_{\mathrm{eff}} & ={\left(\frac{1}{n_{A}}+\frac{1}{n_{B}}\right)}^{-1}\\ & =\frac{n_{A}n_{B}}{n_{A}+n_{B}}. \end{array}$$

**Figure 5:**
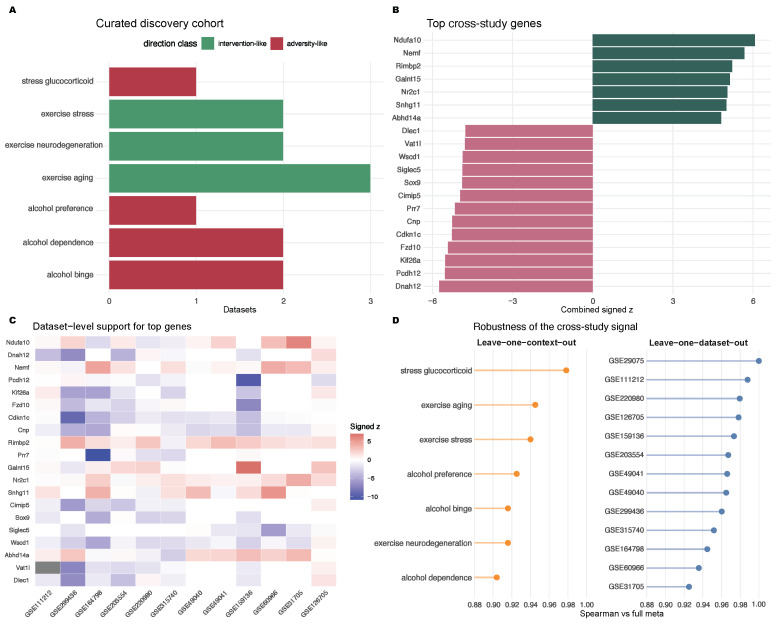
Cross-study discovery cohort definition, harmonization, and robustness of the shared brain transcriptomic signal. (**A**) Composition of the curated bulk discovery cohort used for cross-study integration, grouped by biological context and operational direction class (intervention-like vs adversity-like). Datasets shown are those meeting the predefined inclusion criteria and retained for the weighted cross-study signed *z*-score aggregation. (**B**) Top genes from the weighted cross-study signed *z*-score aggregation, shown with their combined signed *z*-scores after mapping study-specific features to a shared gene–symbol layer. (**C**) Dataset-level support matrix for the top-ranked genes, showing signed differential-expression support across the curated discovery cohort after feature harmonization. (**D**) Robustness of the integrated signal under leave-one-dataset-out and leave-one-context-out analyses, summarized as the Spearman correlation between recomputed combined signed *z*-scores and the full cross-study directional summary. Bulk datasets were analyzed within study, mapped to a shared gene–symbol layer, and combined by effective-sample-size-weighted signed *z*-score aggregation. Robustness was assessed by repeating the aggregation after removing one dataset or one biological context at a time.

The resulting $n_{\mathrm{eff}}$ was used as a weighting quantity rather than as the total sample size. For each harmonized gene, we summarized the number of supporting datasets, weighted mean and unweighted median log_2_ fold change, combined signed *z*-score, concordance of direction across studies, aggregation *p* value, and Benjamini–Hochberg FDR [49]. The gene list shown in Figure 5 was restricted to genes represented in at least two datasets.

This weighted cross-study signed *z*-score aggregation is a representation-level directional summary, not an estimate of a common biological effect size. We did not estimate between-study variance components in the primary aggregation because the studies differ not only in sampling variance but also in species, region, perturbation design, disease background, and assay type. Accordingly, the combined signed *z*-score should be interpreted as weighted cross-study directional evidence and not as an unbiased estimate of one shared effect size or as a fully specified random-effects meta-analytic statistic. The corresponding aggregation *p* values and FDRs were used for ranking and screening within this signed-effect representation; they should not be interpreted in the same way as confirmatory *p* values or FDRs from a homogeneous-effect meta-analysis.

As the principal cross-species robustness experiment, we repeated the primary representation analysis after restricting mouse and rat features to formally defined one-to-one orthologs. Mouse–rat orthology relationships were retrieved from Ensembl BioMart (EMBL-EBI, Hinxton, UK) release 116. We retained only records classified as ortholog_one2one with an Ensembl orthology-confidence score of 1 and non-missing Ensembl gene identifiers and official gene symbols in both species. Bidirectional uniqueness was additionally enforced at the symbol level: each retained mouse symbol was required to correspond to exactly one rat symbol, and each retained rat symbol was required to correspond to exactly one mouse symbol. One-to-many, many-to-many, paralogous, low-confidence, symbol-ambiguous, and species-specific relationships were excluded without selecting representative mappings. Rat genes were represented by the official symbol of their unique mouse ortholog, whereas mouse genes retained their official mouse symbols. This procedure yielded 14,559 high-confidence, bidirectionally unique mouse–rat ortholog pairs.

The ortholog-restricted reanalysis used the same accession-level differential-expression estimates, dataset inclusion rules, predefined intervention-like and adversity-like direction classes, effective-sample-size weights, and weighted signed-*z* aggregation as the primary analysis. Where multiple within-dataset features mapped to the same retained reference gene, log_2_ fold change was summarized by the median and signed-*z* evidence was recomputed across the mapped features before cross-study aggregation. We then recomputed the transcriptome-wide intervention-like versus adversity-like correlation, 1000 dataset-label permutations, reciprocal-gene ranking, selection of the top 100 reciprocal genes, and Ward minimum-variance hierarchical clustering. Candidate k=2, k=3, and k=4 solutions were compared using average silhouette width. Concordance with the original analysis was quantified using the Spearman correlation of reciprocal scores, top-100 overlap and Jaccard index, adjusted Rand index, and module-specific Jaccard indices among genes represented in both solutions. This analysis tested sensitivity to the cross-species mapping strategy; it was not treated as an independent dataset-level replication.

To test whether the integrated discovery signal was disproportionately driven by any individual study or context, we performed leave-one-dataset-out and leave-one-context-out sensitivity analyses. In leave-one-dataset-out analyses, each successfully analyzed discovery dataset was removed in turn and the harmonized gene-level aggregation was recomputed from the remaining studies. In leave-one-context-out analyses, all datasets belonging to a given biological context were removed together before recomputation. For each rerun, we compared the resulting gene-level signal with the full cross-study directional summary using the Spearman correlation [50] of combined signed *z*-scores across shared genes and by calculating overlap among the top 100 ranked genes. Context-stratified summaries additionally tracked mean signed effects within intervention-like and adversity-like direction classes. These robustness analyses, together with directional concordance across contributing datasets, were treated as essential for interpreting the weighted signed *z*-score results.

### 4.5. Operational Modeling of the Reciprocal Representation

**Figure 6:**
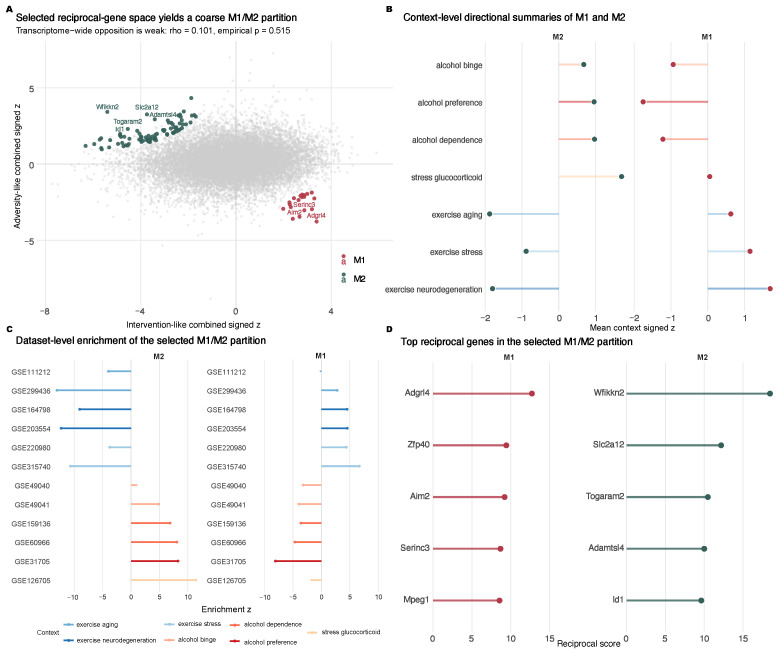
Representation benchmarking and coarse module 1/module 2 (M1/M2) discretization of reciprocal genes. (**A**) Transcriptome-wide intervention-like versus adversity-like scatter for all shared genes, with highlighted points marking genes assigned to the coarse M1/M2 discretization. The highlighted reciprocal genes occupy two counter-directed quadrants relative to the broader background cloud. (**B**) Context-level mean signed effects for M1 and M2 across biological contexts. Intervention-like contexts show positive M1 and negative M2 means, whereas adversity-like contexts show the opposite pattern. (**C**) Dataset-level enrichment of the coarse M1/M2 discretization across the curated discovery cohort, shown as signed enrichment *z*-scores. Larger points denote datasets meeting the enrichment false discovery rate (FDR) threshold. (**D**) Top reciprocal genes used to construct the selection-conditioned coarse M1/M2 discretization, ranked by reciprocal score. Panels A–D summarize the scatter, context-level means, dataset-level enrichment, and representative genes for the coarse M1/M2 discretization. In the primary-cohort analysis, M1 contains 19 genes and M2 contains 81 genes. Because this partition was derived after reciprocal-gene selection, the opposite M1/M2 directions should be read as descriptive structure within the selected space rather than as an independent transcriptome-wide discovery.

For the primary analysis of the reciprocal representation summarized in Figure 6, the harmonized primary bulk discovery datasets were partitioned according to the prespecified intervention-like and adversity-like direction classes defined above. Exercise-trait datasets were excluded from this primary analysis of the reciprocal representation. Separate group-level cross-study directional summaries were then computed for these two classes using the same weighted signed *z*-score framework applied in Figure 5. The two direction classes defined the signed summary space in which the competing representations were evaluated; they were not treated as an established biological dichotomy. The global-axis and reciprocal models were therefore considered alternative descriptions conditional on this prespecified contrast orientation. We assessed whether the resulting structure exceeded that obtained under class-label permutation, remained interpretable at the individual-context level, and persisted after removal of individual datasets or contexts and adjustment for major structural sources of heterogeneity. These analyses were intended to determine the robustness and limits of the operational grouping, rather than to establish a mechanistic dichotomy among the included contexts.

We compared two operational representations conditional on the prespecified contrast orientation. The global-axis model predicted broad inverse alignment between intervention-like and adversity-like combined signed *z*-score summaries. The reciprocal model predicted that any shared opposition would be concentrated in a structured low-dimensional subspace, rather than distributed across the transcriptome. To test the global-axis model, we first calculated the Spearman correlation [50] between intervention-like and adversity-like combined signed *z*-score values across all shared genes. We then compared the observed statistic with an empirical null distribution generated by random reassignment of datasets to the two direction classes while preserving group sizes. We also compared linear and non-linear fits in the same summary space to test whether simple correlation missed a hidden global-axis relationship.

To operationalize the reciprocal alternative, we focused on genes with the strongest and most consistent counter-directed behavior across intervention-like and adversity-like summaries. Genes were ranked by the product of intervention-like and adversity-like signed effects after requiring opposite overall directions and support from at least two datasets in each class. This ranking step was not intended to define a canonical gene list. It provided a working approximation to the region of transcriptomic space most inconsistent with the global-axis model. For each selected gene, we extracted its dataset-level signed *z*-score profile across the full discovery cohort. We row-centered and scaled these profiles, then performed hierarchical clustering using Ward’s minimum-variance method [51]. Candidate solutions with k=2, k=3, and k=4 clusters were compared using average silhouette width. The silhouette-selected two-module solution was retained as a coarse M1/M2 discretization of the selected reciprocal-gene space. Because clustering was performed only after reciprocal-gene selection, the M1/M2 partition was treated as a selection-conditioned descriptive discretization rather than as an independent test of transcriptome-wide reciprocal organization. The opposite directions of M1 and M2 are therefore partly induced by the reciprocal-gene selection rule and were not used as independent evidence for the existence of reciprocal organization. M1 and M2 labels were assigned post hoc for interpretability according to the dominant direction of the two cluster centroids; reversing the labels would not affect the underlying analyses. Because the reciprocal representation is expected to remain partly continuous, this two-module solution was interpreted as an operational approximation to the selected space, not as a uniquely identified latent decomposition.

To quantify how the coarse M1/M2 discretization behaved within individual datasets and broader biological contexts, we computed enrichment scores using permutation-matched background gene sets. For a given dataset or context, the observed score was the mean signed *z*-score value across genes in that partition. We generated 1000 null scores from randomly sampled gene sets of identical size drawn from the shared background gene universe. From this null distribution, we calculated an enrichment *z*-score and empirical two-sided *p* value. FDR was controlled within each partition using the Benjamini–Hochberg procedure [49]. Dataset-level enrichment was computed separately for each accession. Context-level enrichment used mean signed *z*-score values aggregated by biological context before applying the same permutation framework. These analyses quantify directional concentration of signal within the coarse M1/M2 discretization. They are not, by themselves, a formal reproducibility analysis of membership.

We therefore added three formal rigor analyses. First, we tested whether a one-dimensional axis remained adequate after allowing non-linearity. We compared five-fold out-of-sample prediction error for linear and LOESS models of the adversity-like signed *z*-score as a function of the intervention-like signed *z*-score across all shared genes. We also summarized the fraction of variance explained by the first principal component of the dataset-profile matrix used to define the reciprocal genes. To compare a rank-1 latent profile alternative with the coarse M1/M2 discretization, we performed a five-fold gene-wise out-of-sample reconstruction assessment within the top reciprocal-gene profile matrix. This compared reconstruction error under a rank-1 latent profile model with error under a hard two-centroid M1/M2 model fitted only on training genes. This comparison was interpreted as a reconstruction–interpretability trade-off, not as evidence that the M1/M2 partition has a reconstruction advantage over the simpler latent profile. Second, we assessed conditional discretization behavior across top-gene cutoffs of 50, 75, 100, 125, and 150 genes and across Ward, average, and complete linkage. For each run, we compared average silhouette width across k=2, k=3, and k=4 clusters. For each two-module solution, we quantified agreement with the reference partition using the Jaccard index and adjusted Rand index. Third, we performed 100 bootstrap resamples of the discovery datasets within intervention-like and adversity-like classes. We reran the same M1/M2 approximation with a fixed two-module solution and summarized each reference gene by selection frequency and assignment consistency conditional on selection. Selection frequency measures how often a reference gene re-entered the selected reciprocal-gene set, whereas assignment consistency measures whether a re-selected gene was assigned to the same directional side. Thus, high conditional assignment consistency does not compensate for low selection frequency or establish reproducible membership. These analyses were designed to separate conditional two-direction geometry from the reproducibility of exact gene membership.

To probe structured heterogeneity more directly, we performed an additional dataset-level sensitivity analysis using only studies with complete covariate information. We modeled dataset-level M1 and M2 scores as weighted outcomes. Direction class was the coefficient of interest. We first fit this term alone, and we then added structural indicators for species, assay platform, and hippocampal versus non-hippocampal sampling. The final model also included two study-level nuisance summaries: the first principal component of endothelial, microglial, and neuronal proxy scores, and a generic-stress proxy derived from immediate-early and heat-shock response genes. As an approximate hierarchical complement, we fit random-effects meta-regression models to the same module scores. These models treated the dataset-level module statistic as the response and used the same structural and proxy variables as moderators. Because the module statistics are standardized summary scores, these random-effects models should be interpreted as sensitivity analyses for structured heterogeneity. They are not definitive effect-size meta-analyses. We then assessed transferability by re-deriving the two-module solution after leaving out one structural stratum at a time: mouse, rat, RNA-seq, microarray, hippocampal, or non-hippocampal. The resulting partition was compared with the reference partition using Jaccard overlap, the adjusted Rand index, and held-out directional concordance. When the training subset could not support a two-module solution, the corresponding transferability estimate was reported as not estimable.

To model structured heterogeneity at the repeated-observation level, we also fit gene-by-dataset hierarchical mixed models on the reciprocal-gene matrix underlying Figure 6. For each gene–dataset observation, we defined an aligned signed *z*-score statistic. This multiplied the observed signed *z*-score value by the sign expected under the coarse M1/M2 discretization. Positive values therefore indicated directional agreement with the reciprocal representation, regardless of whether the observation belonged to M1 or M2. We fit a Gaussian mixed model for aligned signed *z*-score and a binomial mixed model for sign concordance. Both models included M1/M2 partition label, species, assay platform, hippocampal versus non-hippocampal region group, and biological context as fixed structural terms. Both also included random intercepts for dataset and repeated gene identity. Sum-to-zero contrasts were used for fixed factors so that the intercept corresponded to the grand mean across structural levels. From these models, we derived covariate-adjusted marginal means for M1 and M2 and used Wald- or simulation-based intervals to summarize uncertainty. These hierarchical models tested whether reciprocal alignment persisted after simultaneous adjustment for species, region, platform, and context. They were not intended to establish a causal or fully transportable biological effect.

Downstream human, cellular, spatial, and chromatin analyses were conducted as region-specific evaluation, localization, or contextual projection using fixed M1/M2 definitions. These analyses were not used to redefine module membership or to test exact module reproducibility.

### 4.6. Reference-Atlas Anchoring and Exploratory Single-Nucleus Localization

To localize the reciprocal representation, we first used a preprocessed single-cell reference atlas derived from GSE115746 [25]. Subclass-level reference profiles were collapsed into six broad cell classes (astrocyte, endothelial, excitatory, inhibitory, microglia, and oligodendrocyte) by averaging expression across subclasses sharing the same coarse label. For each side of the M1/M2 discretization, we then calculated mean expression across the corresponding genes within each broad class and converted the resulting profile into a relative *z*-score across classes. This step was used to identify the dominant broad cell classes onto which each side of the discretization projected.

We next performed a lightweight single-nucleus follow-up using GSE237885 [52], which provides four 10x Genomics-style matrices (10x Genomics, Pleasanton, CA, USA) corresponding to WT rest, WT exercise, and ${\mathrm{APP}}^{NL-G-F}$ rest and exercise hippocampal nuclei. Raw matrices were read directly from the GEO tar archive without permanent extraction to disk. Counts were normalized within each sample to log1p counts per 10,000. Broad cell-class labels were assigned to individual nuclei using a marker-based classifier derived from the same reference atlas: for each broad class, the top 50 reference-specific genes were selected by one-versus-rest specificity, and each nucleus was labeled by the broad class with the highest mean marker-expression score.

Within each sample, M1 and M2 module scores were computed for each nucleus by averaging gene-wise standardized expression values across genes belonging to M1 or M2. Because GSE237885 contains one matrix per condition rather than repeated biological samples within condition, this step was interpreted as a localization and qualitative consistency analysis rather than as a formal reproducibility dataset for inference. We therefore summarized mean module scores by inferred broad cell class and sample, and additionally reported exercise-minus-rest score differences within genotype as descriptive follow-up quantities.

### 4.7. Descriptive Pathway Interpretation

To provide a descriptive projection layer for the reciprocal representation, we analyzed the M1/M2 gene sets defined in Figure 6 using a descriptive enrichment workflow rather than treating this step as a primary discovery analysis. M1 and M2 genes were converted to human-style uppercase symbols and mapped to Entrez identifiers with org.Hs.eg.db (Bioconductor Project, Buffalo, NY, USA); over-representation analyses were then performed with clusterProfiler (Bioconductor Project, Buffalo, NY, USA) [53] for Gene Ontology (GO; Gene Ontology Consortium, Hinxton, UK) biological-process terms, Hallmark gene sets, Reactome pathways (Reactome Consortium, Toronto, ON, Canada), and transcription-factor target sets from the Molecular Signatures Database (MSigDB; Broad Institute, Cambridge, MA, USA), including the Gene Transcription Regulation Database (GTRD; BIOSOFT.RU, LLC, Novosibirsk, Russia) collection. Because the coarse M1/M2 discretization was modest in size and originated from cross-species harmonization, internal enrichment cutoffs in clusterProfiler were set permissively and all output terms were retained for manual ranking by FDR, nominal *p* value, and gene-ratio support.

In parallel, we created a focused list of communication-interface genes by flagging module members whose official gene names contained receptor-, ligand-, cytokine-, chemokine-, adhesion-, Notch-, Wnt-, integrin-, cadherin- or semaphorin-related keywords. This layer was intended to identify plausible signaling and intercellular-interface genes embedded within M1 and M2, rather than to claim a full ligand–receptor inference analysis.

### 4.8. Exploratory Human Alcohol Use Disorder Single-Cell Mapping

To evaluate whether the selection-conditioned M1 and M2 scores could be projected in a human alcohol use disorder (AUD) single-cell context, we analyzed readable objects from GSE277313 [54], a caudate-nucleus multiomic dataset from individuals with AUD and controls. Several downloaded files in this accession were truncated, so only objects that passed direct gzip integrity checks (GNU Project, Free Software Foundation, Boston, MA, USA) and could be read successfully through the local workflow were retained for downstream analysis. Because these objects were stored as double-compressed R data-serialization (RDS) files, they were read through a custom streaming decompression helper rather than by full on-disk extraction.

For each retained object, we accessed Seurat object attributes (Satija Lab, New York Genome Center, New York, NY, USA) [16,34] directly to avoid dependence on unavailable local Seurat packages. Cell-level metadata were extracted from the embedded meta.data attribute, which included disease classification. RNA assay matrices were obtained from the stored assay list, and normalized RNA expression values were used for scoring. Broad cell-class labels were assigned using the same marker-based framework defined for the single-nucleus localization analysis after converting both reference markers and feature names to uppercase symbols. Reciprocal-module scores were then computed per cell by averaging gene-wise standardized expression values across the genes in M1 or M2. We summarized these scores both overall and by inferred broad cell class for each readable object, and we reported the AUD-minus-control deltas only for objects containing both case and control cells with adequate counts. Because the available human objects represented multiple pools, varied diagnostic subgroup structure, and marker-based class transfer from mouse reference labels, this analysis was interpreted as exploratory score projection and localization rather than as a formal pooled human reproducibility test.

### 4.9. Region-Specific Human Bulk Evaluation, Specificity Controls, and Chromatin Contextualization

For the prespecified secondary human bulk evaluation, we analyzed GSE181804 [55], a bulk human hippocampus dataset with AUD and control samples. Counts were normalized to log-CPM after TMM scaling [56], mapped to human gene symbols, and scored for M1, M2, and the reciprocal index (M2 minus M1). We additionally derived endothelial, microglial, neuronal, and generic-stress proxy scores by averaging standardized expression across curated marker sets. Endothelial, microglial, and neuronal proxy sets were taken from the top broad-class markers used in the reference-atlas analysis, whereas the generic-stress proxy was defined from canonical immediate-early and heat-shock response genes. AUD effects were estimated using linear models with standard sample-level covariates, first in a base model and then in an adjusted model including endothelial and stress proxy scores.

For secondary cross-region human evaluation, we applied the same M1, M2, and reciprocal-index scoring framework to GSE253155 [57] bulk AUD samples from DLPFC and nucleus accumbens, again testing AUD effects before and after endothelial and stress adjustment. We also re-summarized the GSE277313 single-cell resource within broad cell classes by calculating AUD-minus-control deltas for M1 and M2 separately within each readable object and then aggregating these class-level shifts across objects. This allowed us to distinguish within-class expression shifts from simple case–control differences in cell abundance. Because the human datasets represented different brain regions and produced non-uniform effects, these analyses were treated as dataset- and brain-region-stratified evaluations rather than as a single pooled reproducibility test of the reciprocal representation. We therefore did not interpret a single-region or single-partition association as evidence for the full M1/M2 representation across human brain regions.

To test whether the reciprocal representation merely recapitulated known marker programs, we quantified the overlap of the M1/M2 discretization with top endothelial, microglial, and neuronal marker sets, as well as with a curated generic-stress response set, using Fisher’s exact test [58] against the shared gene universe used to define the M1 and M2 modules in Figure 6. We additionally quantified human asymmetry by re-fitting adjusted linear models for M1, M2, and the reciprocal index across all available bulk human datasets and then pooling the resulting AUD coefficients by inverse-variance weighting, while reporting Cochran’s *Q* and $I^{2}$ as heterogeneity summaries [59,60]. Finally, to provide a chromatin projection layer, we reused the separate exercise chromatin accession GSE208633 [61] and ranked genes in the reciprocal representation by promoter-level H3K27me3 and H4K8ac exercise-minus-sedentary changes. This chromatin layer was treated as correlational contextualization of regulatory alignment, not as evidence of reproducibility, causality, or mechanism.

### 4.10. Coordinate-Aware Anatomical Spatial Localization

To provide a tissue-image spatial localization view of the M1 and M2 modules, we added GSE264692 [62], a human hippocampus spatial atlas generated with 10x Genomics Visium [44] with downloadable histology images, position tables, and scale factors. We selected one representative section (GSM8226201_V11L05-333_A1) and loaded its matrix, feature table, barcode list, tissue-position table, and histology image directly from the GEO sample-level files. Spot matrices were normalized to log1p counts per 10,000, restricted to genes present in the M1 and M2 modules defined in Figure 6 and standardized gene-wise within the section so that module scores reflected relative spatial enrichment across the captured tissue rather than between-study directionality.

For each in-tissue spot, M1 and M2 scores were defined as the mean standardized expression across the valid genes in that module. Coordinate values from the tissue-position file were scaled to the supplied high-resolution tissue image using the accompanying scalefactors_json file, allowing direct overlay of spot-level module values on the histology image. To aid interpretation, we also selected one representative gene per module by ranking module genes according to their within-section correlation with the corresponding module score; this yielded *SERINC3* for M1 and *SNCG* for M2 in the representative section. This analysis was interpreted as anatomical localization of fixed M1 and M2 scores in an external reference tissue rather than as directional evidence or a test of module reproducibility.

### 4.11. Exploratory Spatial Transcriptomic Follow-Up Without Anatomical Reconstruction

To add a spatially sampled extension to the coarse M1/M2 discretization framework, we analyzed GSE271564 [63], the spatial counterpart to the aging-exercise dataset family. The local archive contained three 10x Genomics-style spot matrices corresponding to old exercise, old control, and young control samples, together with feature annotations and barcode identities. Because tissue-coordinate and histology-image files were not present in the downloaded GEO archive, we did not attempt anatomical reconstruction of the captured sections.

Instead, we performed a barcode-matched spot-level analysis. Spot matrices were normalized to log1p counts per 10,000, restricted to genes belonging to the M1 and M2 modules defined in Figure 6, and then pooled across all three samples to compute gene-wise standardized expression values. M1 and M2 scores were defined for each spot as the mean standardized expression across module genes. We summarized these spot scores by sample, compared old exercise against old control across barcode-matched array slots, and tabulated old exercise minus old control changes for individual module genes using sample-level mean normalized expression.

## 5. Conclusions

This benchmark refines, rather than confirms, a simple adaptive-versus-adverse narrative. Across prespecified intervention-like and adversity-like summaries, the assembled data did not support a transcriptome-wide global inverse axis; instead, reciprocal structure was concentrated in a selected counter-directed gene subset. Within this subset, the rank-1 latent profile was the stronger reconstruction model, whereas M1/M2 served as an interpretable directional summary whose human signal was most evident for hippocampal M1 and whose cellular, spatial, and chromatin projections were contextual rather than confirmatory.

Future studies should preregister fixed gene definitions, scoring rules, and primary endpoints, and then test them in independent, preferably homogeneous cohorts matched by species, brain region, platform, perturbation, and sampling design. Prospective work should directly compare continuous latent profiles with discrete M1/M2 scores and use independent human hippocampal and multi-region cohorts plus controlled exercise–alcohol or exercise–stress experiments to assess reproducibility and mechanism.

## Figures and Tables

**Table 1:** Unique Gene Expression Omnibus (GEO) accessions and analysis rows that contributed directly to the analyses reported in this study.

Accession	Species	Brain Region	Data Type	Comparison or Material	Sample Size	Analysis Role
GSE299436	Rat	Hippocampus	Bulk RNA-seqcounts	Old exercise vs. old sedentary	4 vs. 4	Primary discovery(intervention-like)
GSE111212	Mouse	Hippocampus	Bulk RNA-seqcounts	20-month exercise vs. control	5 vs. 5	Primary discovery(intervention-like)
GSE29075	Mouse	Hippocampus	Microarray	Aged running vs. aged sedentary	5 vs. 5	Primary discovery(intervention-like)
GSE220980	Mouse	Medial prefrontalcortex	Normalized bulkRNA-seq	Running vs. stress-model group	3 vs. 3	Primary discovery(intervention-like)
GSE315740	Mouse	Hippocampus	Bulk RNA-seqcounts	Exercise vs. CSDS-model group	6 vs. 6	Primary discovery(intervention-like)
GSE164798	Mouse	Brain	Bulk RNA-seqcounts	WT acute exercise vs. sedentary	4 vs. 4	Primary discovery(intervention-like)
GSE203554	Mouse	Dentate gyrus	Bulk RNA-seqcounts	APP/PS1 model exercise vs. no exercise	10 vs. 5	Primary discovery(intervention-like)
GSE159136	Rat	Central amygdala	Bulk RNA-seqcounts	Chronic intermittent ethanol vs. air control	6 vs. 6	Primary discovery(adversity-like)
GSE49040	Rat	Central amygdala	Microarray	Ethanol vs. water	10 vs. 10	Primary discovery(adversity-like)
GSE49041	Rat	Nucleus accumbensshell	Microarray	Ethanol vs. water	10 vs. 10	Primary discovery(adversity-like)
GSE60966	Rat	Multiple brainregions	Microarray	Alcohol-dependent vs. control	32 vs. 32	Primary discovery(adversity-like)
GSE31705	Rat	Nucleus accumbensshell	Microarray	Alcohol-preferring vs. non-preferring	49 vs. 48	Primary discovery(adversity-like)
GSE126705	Rat	Hippocampus	RNA-seq exoncounts	FS60 vs. early-morningbaseline	6 vs. 4	Primary discovery(adversity-like)
GSE115746	Mouse	Brain referenceatlas	Single-cell referenceprofiles	Preprocessed subclass expression profiles	Reference profiles	Exploratory reference-atlasanchoring
GSE237885	Mouse	Hippocampus	Single-nucleusRNA-seq	WT/APP by rest/exercise	4 matrices; 38,636 nuclei	Exploratory single-nucleuslocalization
GSE277313	Human	Caudate nucleus	Single-cell multiome	AUD vs. controlwithin readable objects	9 paired objects; 104,896 cells	Exploratory human cell-stateprojection
GSE181804	Human	Hippocampus	Bulk RNA-seqcounts	AUD vs. control	12 vs. 12	Secondary human bulkevaluation
GSE253155	Human	DLPFC	Bulk RNA-seqcounts	AUD vs. control	38 vs. 35	Secondary cross-regionhuman evaluation
GSE253155	Human	Nucleus accumbens	Bulk RNA-seqcounts	AUD vs. control	42 vs. 38	Secondary cross-regionhuman evaluation
GSE208633	Mouse	Hippocampus	Chromatin profiling	Exercise vs. sedentary promoter marks	Promoter-level peaks	Exploratory chromatincontextualization
GSE264692	Human	Hippocampus	Spatialtranscriptomics	Representative Visium section(10x Genomics, Pleasanton, CA, USA)	4992 in-tissue spots	Exploratory anatomicalspatial localization
GSE271564	Mouse	Aging brain	Spatialtranscriptomics	Young control, old control, and old exercise	3 samples; 4992 spots each	Exploratory exercise–agingspatial follow-up

**Notes:** The final analyses included 21 unique GEO accessions represented by 22 analysis rows. Abbreviations: GEO, Gene Expression Omnibus; CSDS, chronic social defeat stress; WT, wild-type; APP, amyloid precursor protein; PS1, presenilin 1; AUD, alcohol use disorder; DLPFC, dorsolateral prefrontal cortex; FS60, 60 min after forced swim onset; M1/M2, module 1/module 2; RNA-seq, ribonucleic acid sequencing. The first 13 accessions constituted the primary weighted discovery cohort. Evaluation, reference, localization, and projection accessions were analyzed separately and were not used to define the primary M1/M2 partition. GSE253155 is shown in two rows because DLPFC and nucleus accumbens samples were modeled as separate brain-region analyses.

## Data Availability

The public accessions used for primary discovery, human evaluation, cellular localization, spatial analysis, and chromatin projection are listed in Table 1. The 21 unique GEO accessions that contributed directly to this study are available from the NCBI Gene Expression Omnibus (GEO). Direct GEO record links for all accessions are provided in accession-and-URL format below. All URLs were accessed on 3 April 2026: GSE299436, https://www.ncbi.nlm.nih.gov/geo/query/acc.cgi?acc=GSE299436; GSE111212, https://www.ncbi.nlm.nih.gov/geo/query/acc.cgi?acc=GSE111212; GSE29075, https://www.ncbi.nlm.nih.gov/geo/query/acc.cgi?acc=GSE29075; GSE220980, https://www.ncbi.nlm.nih.gov/geo/query/acc.cgi?acc=GSE220980; GSE315740, https://www.ncbi.nlm.nih.gov/geo/query/acc.cgi?acc=GSE315740; GSE164798, https://www.ncbi.nlm.nih.gov/geo/query/acc.cgi?acc=GSE164798; GSE203554, https://www.ncbi.nlm.nih.gov/geo/query/acc.cgi?acc=GSE203554; GSE159136, https://www.ncbi.nlm.nih.gov/geo/query/acc.cgi?acc=GSE159136; GSE49040, https://www.ncbi.nlm.nih.gov/geo/query/acc.cgi?acc=GSE49040; GSE49041, https://www.ncbi.nlm.nih.gov/geo/query/acc.cgi?acc=GSE49041; GSE60966, https://www.ncbi.nlm.nih.gov/geo/query/acc.cgi?acc=GSE60966; GSE31705, https://www.ncbi.nlm.nih.gov/geo/query/acc.cgi?acc=GSE31705; GSE126705, https://www.ncbi.nlm.nih.gov/geo/query/acc.cgi?acc=GSE126705; GSE115746, https://www.ncbi.nlm.nih.gov/geo/query/acc.cgi?acc=GSE115746; GSE237885, https://www.ncbi.nlm.nih.gov/geo/query/acc.cgi?acc=GSE237885; GSE277313, https://www.ncbi.nlm.nih.gov/geo/query/acc.cgi?acc=GSE277313; GSE181804, https://www.ncbi.nlm.nih.gov/geo/query/acc.cgi?acc=GSE181804; GSE253155, https://www.ncbi.nlm.nih.gov/geo/query/acc.cgi?acc=GSE253155; GSE208633, https://www.ncbi.nlm.nih.gov/geo/query/acc.cgi?acc=GSE208633; GSE264692, https://www.ncbi.nlm.nih.gov/geo/query/acc.cgi?acc=GSE264692; and GSE271564, https://www.ncbi.nlm.nih.gov/geo/query/acc.cgi?acc=GSE271564. Thirteen accessions formed the primary bulk discovery cohort, whereas eight accessions were used for human evaluation, cellular or single-nucleus localization, chromatin projection, and spatial follow-up. GSE253155 contributed two analysis rows because DLPFC and nucleus accumbens samples were modeled separately. GSE111212 and GSE271564 are cited as GEO records because no clearly linked peer-reviewed primary article was used. No new datasets were generated in this study.

## Supplementary Materials

The following supporting information can be downloaded at: https://www.mdpi.com/article/10.3390/ijms27136083/s1.
