# Supplementary material for: Systematic Evaluation of Competing Brain Transcriptomic Representations Reveals Reciprocal Patterns Across Heterogeneous Contexts
Source: Int J Mol Sci. 2026 Jul 7;27(13):6083. doi: 10.3390/ijms27136083 (PMC13362261; doi:10.3390/ijms27136083)
Supplement: Supplementary file 1 [file ijms-27-06083-s001.zip › ijms-4367303-supplementary.pdf]

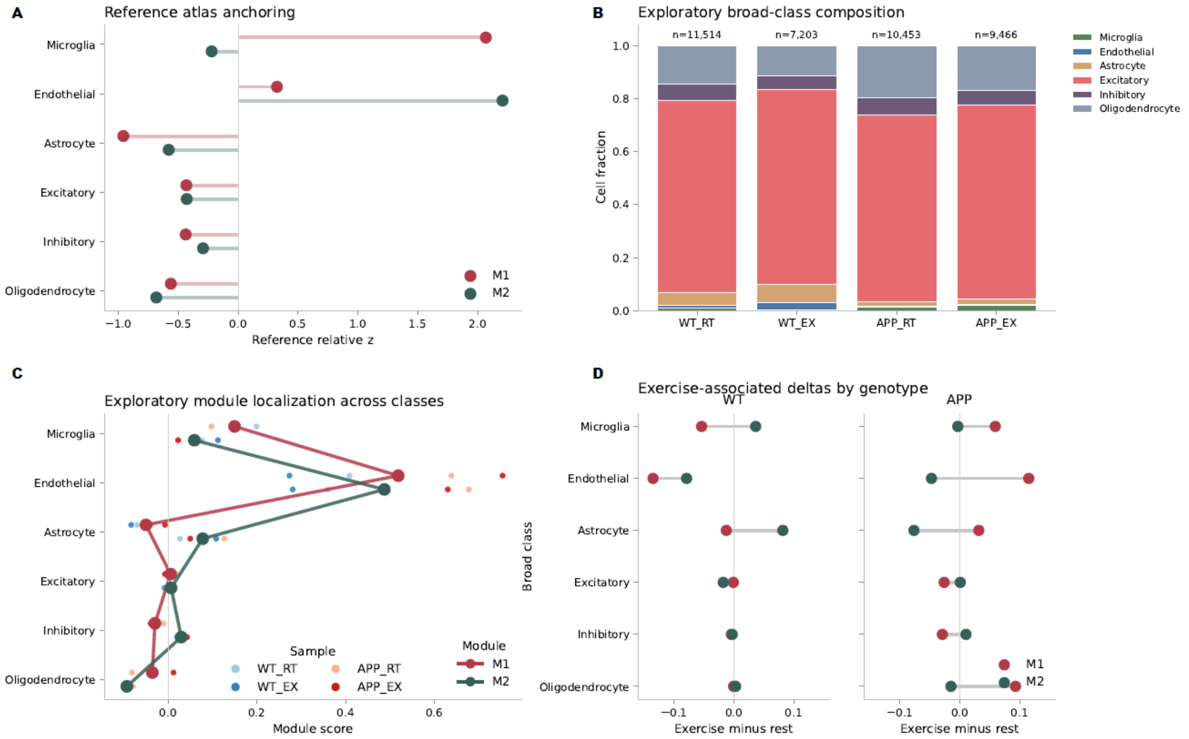

Figure S1. Cell-state localization of the M1 and M2 modules. Panels A–D present reference-atlas anchoring, broad-class composition of GSE237885, projected module scores across inferred broad cell classes, and within-genotype exercise-minus-rest differences.

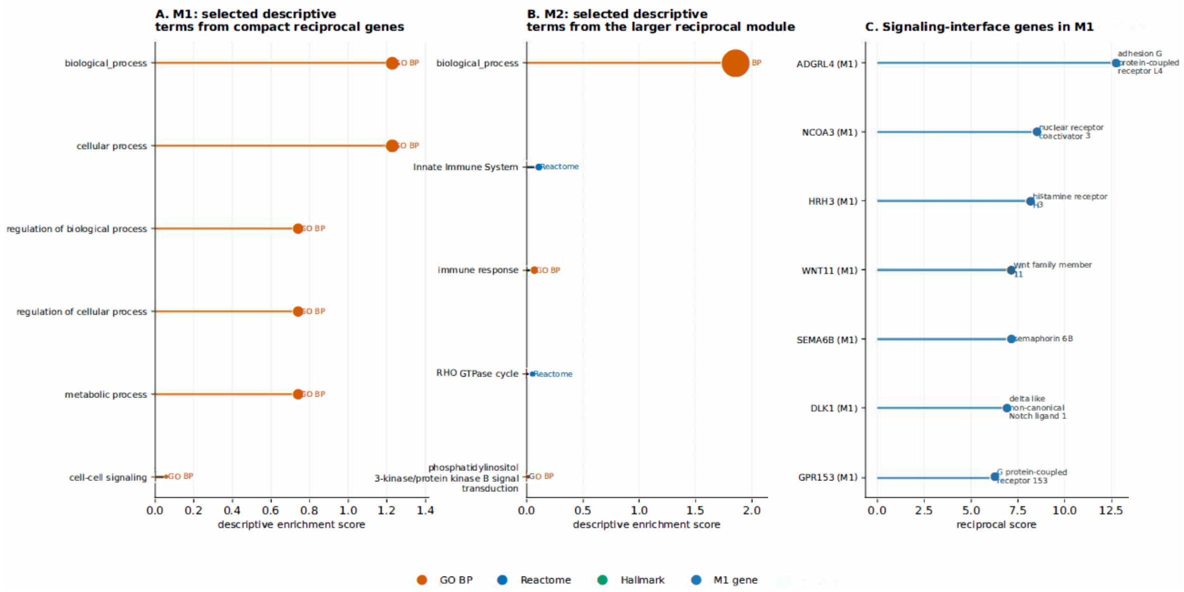

Figure S2. Descriptive pathway and signaling-interface interpretation of the selection-conditioned M1/M2 partition.

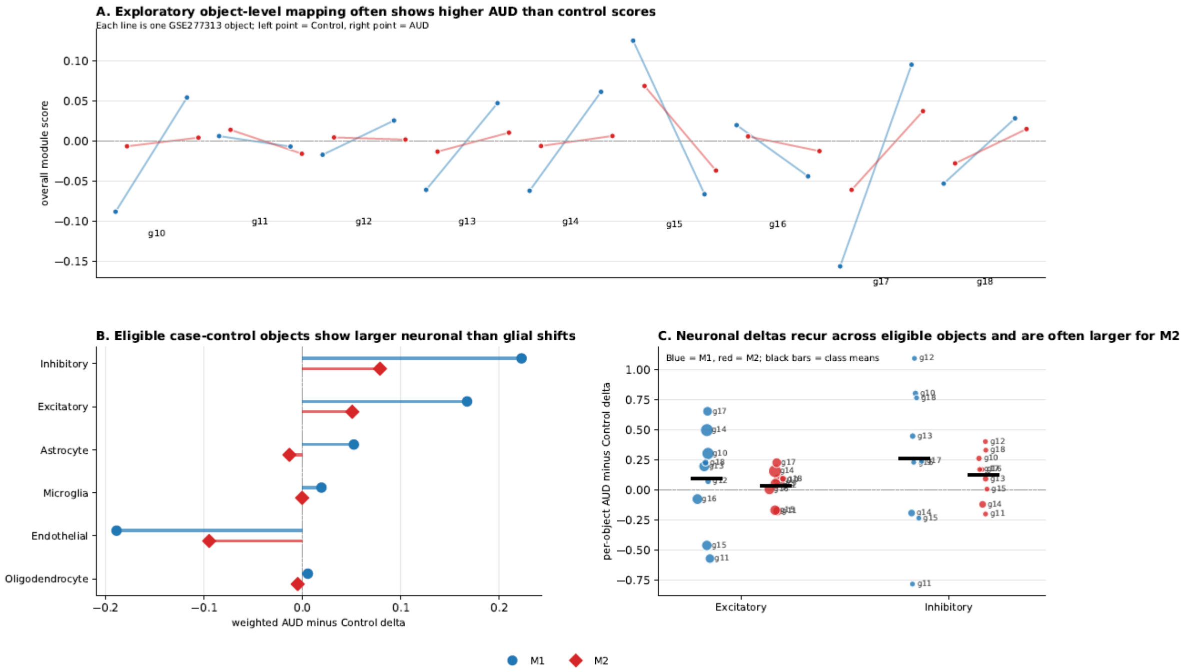

Figure S3. Exploratory object-level and class-resolved M1/M2 score projections in the human caudate AUD dataset GSE277313.

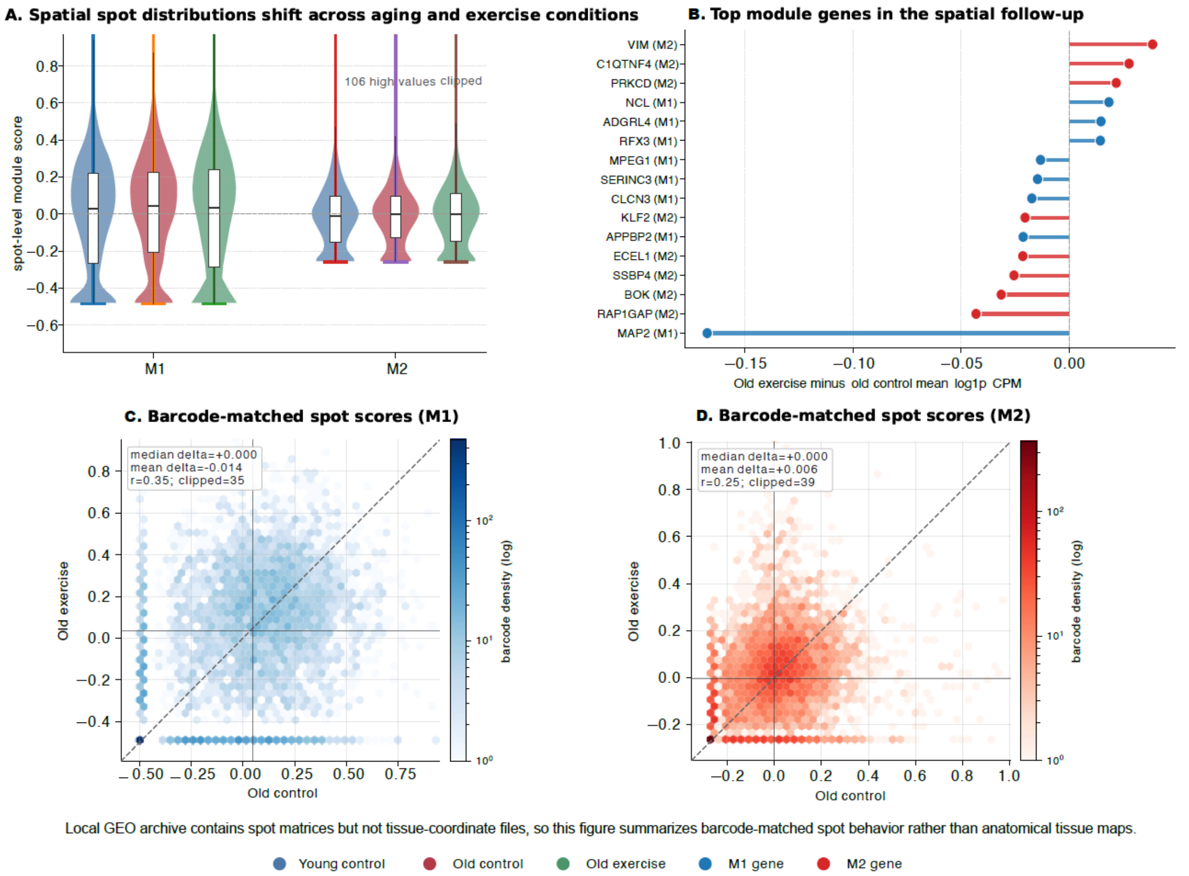

Figure S4. Exploratory spot-level spatial follow-up of M1 and M2 modules in the exercise-aging dataset GSE271564.
